# Apparent total tract nutrient digestibility and metabolizable energy estimation in commercial fresh and extruded dry kibble dog foods

**DOI:** 10.1093/tas/txab071

**Published:** 2021-05-27

**Authors:** Jirayu Tanprasertsuk, LeeAnn M Perry, Devon E Tate, Ryan W Honaker, Justin Shmalberg

**Affiliations:** 1 NomNomNow, Inc., Nashville, TN 37207, USA; 2 Department of Comparative, Diagnostic, and Population Medicine, College of Veterinary Medicine, University of Florida, Gainesville, FL 32611, USA

**Keywords:** canine nutrition, fresh food, crude protein, nitrogen-free extract

## Abstract

Commercial fresh cooked foods have started gaining popularity among American dog owners in recent years. However, nutrient digestibility and the estimation of metabolizable energy (ME) of commercial fresh dog foods remain inadequately understood, even though both measures are critical to provide the intended calories for the target animal. In this preliminary study, different cohorts of normal-weight dogs were fed one of five test diets of comparable macronutrient composition: a chicken-based extruded dry kibble diet (*n* = 12), and chicken- (*n* = 12), beef- (*n* = 6), pork- (*n* = 6), or turkey-based fresh food (*n* = 6) for 10 d. Daily food intake and fecal characteristics were recorded, and fecal samples were collected for nutrient analysis. Despite comparable dry matter (DM) and caloric intakes between the two chicken-based diets, the fresh diet led to lower defecation frequency (1.2 ± 0.2 vs. 1.7 ± 0.5 times/d, adjusted *P* < 0.001), lower fecal DM (24 ± 8 vs. 47 ± 10 g/d, adjusted *P* < 0.001), and lower fecal calories (92 ± 31 vs. 189 ± 43 kcal/d, adjusted *P* < 0.001) than the kibble diet. The apparent total tract digestibility of DM, protein, fat, nitrogen-free extract, and calories of the kibble diet were all significantly lower than any of the fresh diets (adjusted *P* < 0.001 for all). Measured ME per food DM in all of the fresh diets, except the pork-based recipe, was significantly higher than that of the kibble diet (adjusted *P* < 0.001 for all). For the kibble diet, the modified Atwater calculation underestimated the ME and the NRC 2006 calculation was the most accurate predictor of ME. The standard Atwater calculation performed best for the two fresh diets that had the highest fat content (chicken, beef) and the NRC 2006 calculation performed best for the fresh diet that had the highest protein content (pork). ME of the turkey-based diet was equally overestimated and underestimated with the standard Atwater and NRC 2006 methods, respectively. We propose that commercial and home-prepared fresh diets should be assessed using standard Atwater factors as commonly done in human nutrition, or preferably for commercial products, by direct measurement in conforming feeding trials.

## INTRODUCTION

Genetic studies suggest that dogs were domesticated from gray wolves approximately 11,000–16,000 yr ago (Freedman *et al.*, 2014; Fan *et al.*, 2016). Over thousands of years of coevolution, humans have shared starch-rich cultivated foods with dogs, resulting in changes in the canine genome allowing them to digest carbohydrates more efficiently (Axelsson *et al.*, 2013). It was not until the 1860s that the first commercially-prepared dog food in the form of a biscuit composed of wheat meals, vegetables, beet, and beef blood was formulated in England (Schaffer, 2009). Since then commercial dog food, especially in the form of extruded kibble, has become the most popular choice among household dog owners in the United States (Laflamme *et al.*, 2008; Dinallo *et al.*, 2017). Extrusion cooking refers to a process where a mixture of ingredients is steam conditioned, compressed, and forced through the die of an extruder (Tran *et al.*, 2008). It is high-throughput for mass production, and it offers convenience and longer shelf-life for dog owners. However, the high-temperature (≥200 ^o^C), low-moisture (<15%) conditions during extrusion may decrease the digestibility of starch, protein, and fats, and destroy bioactive compounds present in the raw ingredients (Singh *et al.*, 2007; Tran *et al.*, 2008; Moreno *et al.*, 2017).

While dogs have shared food with their human companions in many cultures across the world, it is only in recent years that commercial fresh dog foods have started gaining popularity in the U.S. market. Fresh diets are cooked without extrusion using a variety of techniques including kettle cooking, steam cooking, and the separate cooking of each ingredient followed by blending. It is most commonly refrigerated or frozen and thawed prior to consumption. Commercial fresh dog foods provide increased convenience compared to home cooked foods, and must comply with the Association of American Feed Control Officials (AAFCO) regulations to meet nutrient standards for the labeled lifestage.

Nutrient digestibility and metabolizable energy (ME) of commercial fresh dog foods remain inadequately understood with only two prior published studies. The digestibility of six commercial fresh diets has previously been assessed with the precision-fed cecectomized rooster assay (Oba *et al.*, 2020), and determined for two fresh diets in a canine feeding trial (Algya *et al.*, 2018). Dietary digestibility and ME are important measures in veterinary nutrition, as they are affected by individual components of the food such as the degree of cooking, the macronutrient composition, and the amount of fibers. These two measures are necessary for a formulation of diets that ensures adequate amounts of nutrients and energy for normal growth and maintenance.

The primary purpose of the investigation was to assess and compare the apparent total tract nutrient digestibility (dry matter [DM], protein, fat, nitrogen-free extract [NFE], calorie) and ME of nutritionally-comparable commercial kibble and fresh canine diets with a digestibility trial. Fecal nutrient composition and consistency were also compared between diets. A secondary aim was to compare the AAFCO mandated modified Atwater factors, the standard Atwater factors, and the National Research Council (NRC) 2006 calculation with measured ME to provide guidance for home-prepared and commercial fresh diets (National Research Council, 2006; Calvez *et al.*, 2019).

## MATERIALS AND METHODS

### Care and Use of Animals

All research protocols were approved by an Institutional Animal Care and Use Committee.

### Animals and Study Diets

Twelve normal-weight Beagle dogs of similar size (six males and six females) were fed a chicken-based kibble diet then switched to a chicken-based fresh diet. A different cohort of 18 normal-weight Beagle dogs were divided into three groups of six dogs, and each group of six dogs were either fed a beef-based (three males and three females), pork-based (four males and two females), or turkey-based fresh diet (three males and three females). All dogs were at least 1 yr of age and the feeding period for each test diet lasted 10 d. The chicken-based kibble diet used was a grain-free chicken-based commercial kibble option with a similar anticipated nutrient profile based on the guaranteed analyses to the fresh diets. All four fresh diets were from NomNomNow Inc. (Oakland, CA) and were: Chicken Chow, Beef Mash, Pork Potluck, and Turkey Fare. Ingredients of all fresh diets are listed in **Supplemental Table 1** as well as online (https://www.nomnomnow.com/dog-food-recipes). All the test diets meet the AAFCO’s nutrient standards for adult dogs at maintenance.

The test diet was the sole source of food for each diet period. The feeding amounts were based on the dog’s body condition with an aim to maintain their body weight. The dogs were fed once each day at the same time, and food intake was recorded daily by a technician. The first 5 d of the test were considered an acclimation period. Body weights were recorded on d 1 through 6, and on d 10 of each diet period.

### Diet Analysis

A sample of each test diet was analyzed for nutritional content at Eurofins US (Des Moines, IA) (Table 1). Moisture was determined in a forced draft oven with controlled elevated temperatures at 135 ^o^C for 2 h (AOAC 930.15) (AOAC, 2019). For fat content, each sample was hydrolyzed with HCl. The hydrolyzed sample was extracted in a liquid–liquid extraction with a combination of ethyl and petroleum ethers. The ethers containing the fat were collected, dried, and used to calculate the crude fat in the sample (AOAC 954.02). For crude protein, each sample was placed into the combustion chamber of a protein analyzer, and gas from the combustion was analyzed for nitrogen content and used to calculate protein amount (AOAC 992.15, AOAC 990.03, AOCS Ba 4e−93). For crude fiber (CF), each sample was digested with acid and base. The weight of the residue minus the ash from the residue determined the CF content (AOAC 962.09, AOCS Ba 6–84). For the analysis of ash, two grams of sample was weighted into crucible, dried in an oven, then ashed in a muffle furnace at 600 °C and weighed (AOAC 942.05). NFE was calculated by subtracting fat, protein, CF, and ash from DM.

**Table 1.** Nutrient composition of the study diets

Measure	Kibble-chicken			Fresh-chicken			Fresh-beef			Fresh-pork			Fresh-Turkey		
	FW	DM	g/Mcal ME^a^	FW	DM	g/Mcal ME^a^	FW	DM	g/Mcal ME^a^	FW	DM	g/Mcal ME^a^	FW	DM	g/Mcal ME^a^
**Nutrient (%)**															
Moisture	5.40	–	13.78	72.90	–	530.35	71.60	–	509.36	73.60	–	684.77	71.00	–	502.10
Protein	35.44	37.46	90.44	9.94	36.68	72.31	10.56	37.18	75.12	10.50	39.77	97.69	11.56	39.86	81.75
Fat	18.25	19.29	46.57	8.22	30.33	59.80	7.38	25.99	52.50	3.96	15.00	36.84	7.77	26.79	54.95
NFE	29.76	31.46	75.94	6.62	24.43	48.16	7.93	27.92	56.41	9.24	35.00	85.97	7.13	24.59	50.42
Total dietary Fiber	12.5	13.2	31.9	5.5	20.3	40.0	–	–	–	–	–	–	–	–	–
Crude fiber	3.3	3.5	8.4	0.5	1.8	3.6	0.3	1.1	2.1	0.4	1.5	3.7	0.3	1.0	2.1
Soluble fiber	9.2	9.7	23.48	5.0	18.5	36.4	–	–	–	–	–	–	–	–	–
Ash	7.85	8.30	20.03	1.82	6.72	13.24	2.23	7.85	15.86	2.30	8.71	21.40	2.24	7.72	15.84
Phosphorus	1.04	1.10	2.65	0.26	0.96	1.89	0.33	1.16	2.35	0.40	1.52	3.72	0.30	1.03	2.12
Calcium	1.37	1.45	3.50	0.35	1.29	2.55	0.43	1.51	3.06	0.43	1.63	4.00	0.40	1.38	2.83
Ca:P ratio	1.32	1.32	–	1.36	1.36	–	1.30	1.30	–	1.10	1.10	–	1.34	1.34	–
**Calculated ME density (kcal/kg)** ^**b**^															
Atwater	4,251	4,493	–	1,402	5,174	–	1,404	4,943	–	1,146	4,341	–	1,447	4,989	–
Modified Atwater	3,833	4,051	–	1,278	4,716	–	1,274	4,488	–	1,028	3892	–	1,314	4,533	–

FW: fresh weight, DM: dry matter, NFE: nitrogen-free extract, ME: metabolizable energy (ME calculation in Supplemental Table 1 and ME values in Table 4).

^*a*^ME calculated based on the measured food and fecal energy content with the bomb calorimetry as described in Supplemental Table 1. ME values are reported in Table 4.

^*b*^Atwater and modified Atwater’s ME calculation are explained in Supplemental Table 1.

Additionally, a sample of a chicken-based kibble diet and a chicken-based fresh diet was analyzed for total dietary fiber (TDF) at the Midwest Laboratories (Omaha, NE). Each dried and fat-extracted sample underwent sequential enzymatic digestion by amylase, protease, and amyloglucosidase to remove starch and protein. Enzyme digestate was then treated with alcohol to precipitate soluble fiber before filtering, and TDF residue was washed, dried, and weighed (AOAC 991.43). Soluble fiber was calculated by subtracting CF from TDF.

### Stool Samples and Nutritional Analysis

Defecation frequency, fecal consistency, and adverse effects were recorded from d 6 afternoon to d 11 morning of each diet period. Stool quality was measured and recorded by a research technician according to a photo grading sheet for each defecation during the collection period. Fecal score ranges were defined as 1: watery diarrhea; 1.5: diarrhea; 2: moist, no form; 2.5: moist, some form; 3: moist, formed; 3.5: well formed, sticky; 4: well formed; 4.5: hard, dry; 5: hard, dry, crumbly. Feces collected from d 6 to d 11 were pooled and homogenized for each dog, and analyzed using the methods described above (Eurofins US, Des Moines, IA). Results of the fecal and dietary analyses were used to calculate apparent total tract DM, protein, fat, and caloric digestibility and ME. The calculation is described in **Supplemental Table 2**.

### Statistical Analysis

Data were expressed as mean ± *SD* for continuous variables or *n* (%) for categorical variables. All outcomes were compared between the 5 diets with Kruskal-Wallis tests and pairwise Wilcoxon rank sum tests. Significance level was set at ***α*** = 0.05, and false discovery rate (FDR) adjustment was applied for pairwise comparisons. All analyses were performed in RStudio 1.2.5033.

## RESULTS

### Daily Food and Nutrient Intakes

All dogs completed the study. Daily food and nutrient intakes during each diet period are shown in Table 2. Dogs consumed comparable DM and calories between the two chicken-based diets. However, due to the difference in nutrient profiles between the two study diets, fat and soluble fiber intakes were significantly higher in the chicken-based fresh diet, while NFE and CF intakes were significantly higher in the chicken-based kibble diet period (FDR-adjusted *P* < 0.01 for all).

**Table 2.** Daily food and nutrient intakes (mean ± *SD*) calculated from d 6 to d 10 of each feeding period.

Measure	Kibble (n = 12)	Fresh C (n = 12)	Fresh B (n = 6)	Fresh P (n = 6)	Fresh T (n = 6)	*P* value*
**Daily food intake**						
Daily food intake, in g FW	279±25^a^	895±173^b^	443±78^c^	463±41^c^	428±22^c^	2.47E–07
Daily food intake, in g DM	264±23^a^	243±47^a^	126±22^b^	122±11^b^	124±6^b^	5.31E–06
Daily food intake, in kcal	1,388±123^a^	1,423±275^a^	718±127^b^	593±53^b^	705±36^b^	1.90E–06
**Daily nutrient intake, in g**						
Protein	98.8±8.7^a^	89.0±17.2^a^	46.8±8.3^b^	48.6±4.3^b^	49.4±2.5^b^	3.64E–06
Fat	50.9±4.5^a^	73.6±14.2^b^	32.7±5.8^c^	18.3±1.6^d^	33.2±1.7^c^	3.09E–07
NFE	83.0±7.3^a^	59.2±11.4^b^	35.1±6.2^c,d^	42.8±3.8^c^	30.5±1.6^d^	2.83E–07
Crude Fiber	9.2±0.8^a^	4.5±0.9^b^	1.3±0.2^c^	1.9±0.2^d^	1.3±0.1^c^	1.22E–07
Soluble Fiber	25.6±2.3	44.7±8.6	–	–	–	5.19E–05
Ash	21.9±1.9^a^	16.3±3.1^b^	9.9±1.8^c^	10.6±0.9^c^	9.6±0.5^c^	5.47E–07

FW: fresh weight, DM: dry matter, fresh C: fresh chicken, fresh B: fresh beef, fresh P: fresh pork, fresh T: fresh turkey.

*Kruskal–Wallis rank sum test. Means not sharing the same superscript are significantly different (pairwise Wilcoxon rank sum tests with false discovery rate adjustment).

Different cohorts of dogs were fed the three remaining test diets. Food and caloric intakes were comparable among the beef-, pork-, and turkey-based fresh diets. Due to the difference in nutrient profile, dogs consumed significantly lower fat and higher CF in the pork-based fresh diet than the beef- and turkey-based fresh diets (FDR-adjusted *P* < 0.01 for all).

### Body Weight, Defecation Frequency, Fecal Consistency, and Adverse Effects

Body weight at baseline was not statistically different between the diet groups (**Supplemental Table 3**). Although it was not intended, differential changes in body weight among the diet groups were observed. Body weight change as a percentage during the kibble diet was significantly higher than all of the non-chicken-based fresh diets (FDR-adjusted *P* < 0.05 for all). Body weight change as a percentage during the chicken-based fresh diet was significantly higher than the pork- and turkey-based fresh diets (FDR-adjusted *P* < 0.05 for all).

Defecation frequency during the kibble diet period was 1.7 ± 0.5 times/d, which was significantly higher than all of the other fresh diets (FDR-adjusted *P* < 0.05 for all) (**Supplemental Table 4**). Defecation frequency during the chicken-based fresh diet (1.2 ± 0.2 times/d) was also significantly higher than the turkey-based fresh diet (1.0 ± 0.2 times/d, FDR-adjusted *P* = 0.038). Comparable fecal consistency was observed across all diets, except that the stool during the turkey-based fresh diet was less firm than that during the chicken-based fresh diet (FDR-adjusted *P* = 0.007). One dog had an incidence of diarrhea (fecal consistency score of 1.5) during the kibble period. Two incidences of vomiting (once in two different dogs) were observed during both the kibble diet and the turkey-based fresh diet, and once was observed during each of the chicken-, beef-, and pork-based fresh diets. No constipation (fecal consistency score ≥ 4) was observed in any diet period.

### Daily Fecal Weight and Nutrient Composition

Fecal weight and nutrient composition are reported in Table 3. Despite comparable food DM intake (Table 2), daily fecal DM was significantly lower in the chicken-based fresh diet (24 ± 8 g/d) as compared to the kibble diet (47 ± 10 g/d, FDR-adjusted *P* < 0.001). The total daily fecal energy output was also significantly lower in the chicken-based fresh diet (92 ± 31 kcal/d) than the kibble diet (189 ± 43 kcal/d, FDR-adjusted *P* < 0.001). Fecal energy density per DM measured by the bomb calorimetry in all fresh diets, except the turkey-based, was significantly lower than the kibble diet (FDR-adjusted *P* < 0.05 for all).

**Table 3.** Fecal fresh and dry weight and nutrient composition per fecal dry matter (mean±SD) during each feeding period. Calorie densities are expressed as calculated gross energy density or measured by the Bomb calorimeter.

Measure	Kibble (n = 12)	Fresh C (n = 12)	Fresh B (n = 6)	Fresh P (n = 6)	Fresh T (n = 6)	*P* value*
**Daily fecal weight, in g**						
** **Fresh weight (FW)	149±39^a^	102±46^b^	46±15^c^	43±5^c^	43±5^c^	1.11E–05
** **Dry matter (DM)	47±10^a^	24±8^b^	12±3^c^	11±1^c^	11±1^c^	2.05E–06
**Fecal moisture, in % FW**	68.45±2.32^a^	75.17±4.57^b^	72.53±3.90^b^	74.08±1.86^b^	74.86±1.94^b^	5.56E–04
**Fecal energy**						
** **Calculated energy density, in kcal/g DM	3.41±0.06^a^	3.47±0.14^a^	3.01±0.06^b^	3.06±0.16^b^	3.64±0.14^c^	6.49E–06
** **Measured energy density, in kcal/g DM	4.07±0.06^a^	3.91±0.18^b^	3.32±0.14^c^	3.40±0.18^c^	3.94±0.26^a,b^	1.33E–05
** **Daily fecal energy, in kcal	189±43^a^	92±31^b^	40±10^c,d^	38±3^c^	42±4^d^	7.25E–07
**Fecal nutrient, in % DM**						
** **Protein	30.80±1.87^a^	30.67±1.63^a^	24.66±1.36^b^	25.00±1.62^b^	25.08±1.88^b^	6.46E–06
** **Fat	5.50±0.59^a^	7.30±1.36^b^	6.63±0.60^b,c^	5.64±0.99^a,c^	11.58±1.68^d^	1.69E–05
** **NFE	27.90±3.07	25.25±3.97	24.06±1.87	27.17±3.83	27.23±4.41	0.098
** **Crude fiber	14.0±0.6^a^	10.2±1.0^b^	7.4±1.2^c^	7.0±1.0^c^	4.5±0.9^d^	1.40E–07
** **Ash	21.81±0.93^a^	26.55±2.87^b^	37.29±1.45^c^	35.24±2.81^c^	31.57±2.98^d^	2.52E–07

NFE: nitrogen-free extract, fresh C: fresh chicken, fresh B: fresh beef, fresh P: fresh pork, fresh T: fresh turkey.

*Kruskal–Wallis rank sum test. Means not sharing the same superscript are significantly different (pairwise Wilcoxon rank sum tests with false discovery rate adjustment).

Fecal moisture content in all fresh diets was significantly higher than the kibble diet (FDR-adjusted *P* < 0.05 for all). As expected, fecal nutrient composition per DM (Table 3) reflected the study diet composition per DM to some extent (Table 1). Fecal fat content was significantly lower in the kibble and the pork-based fresh diet, both of which had relatively lower fat content than the other diets. Fecal CF content was significantly highest in the kibble diet which also had the highest CF content.

### Digestibility of DM, Protein, Fat, NFE, and Calorie

**Figure 1.**
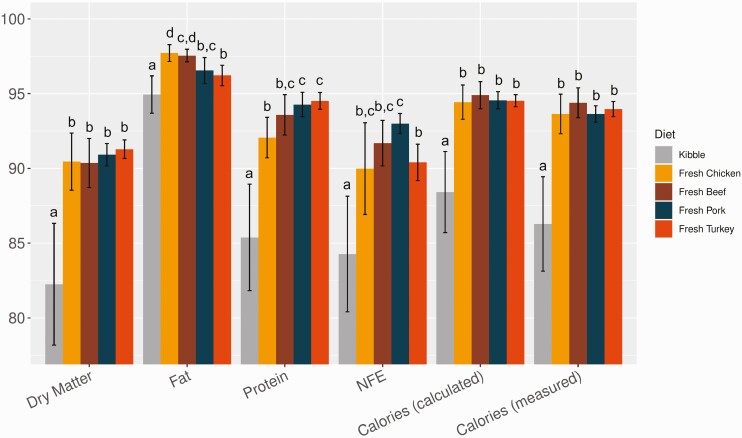
Digestibility (mean ± *SD*, in %) of dry matter, protein, fat, nitrogen-free extract (NFE), and calories in kibble and fresh diets. Calories are expressed as calculated gross energy or measured by the Bomb calorimeter. Means not sharing the same letter are significantly different (FDR-adjusted *P* < 0.05, Kruskal–Wallis rank sum test and post-hoc pairwise Wilcoxon rank sum tests).

As shown in Figure 1, the total (DM) digestibility, protein digestibility, fat digestibility, NFE digestibility, and caloric digestibility of the kibble diet were all significantly lower than any of the fresh diets (FDR-adjusted *P* < 0.001 for all). Among the four fresh diets, total digestibility and calorie digestibility were comparable. The chicken-based fresh diet had significantly higher fat digestibility (FDR-adjusted *P* < 0.05 for both) and lower protein digestibility than the pork- and turkey-based fresh diets (FDR-adjusted *P* < 0.01 for both), and the beef-based fresh diet had higher fat digestibility than the turkey-based fresh diet (FDR-adjusted *P* = 0.011). The pork-based fresh diet also had significantly higher NFE digestibility than the turkey-based fresh diet (FDR-adjusted *P* = 0.010).

### Metabolizable Energy

ME of the five study diets are expressed as kcal/g fresh weight diet, kcal/g DM diet, and %ME/gross energy of food intake, and listed in Table 4. ME were calculated based on either the calculated food and fecal energy content, or the measured food and fecal energy content with the bomb calorimetry. ME per DM in all of the fresh diets, except the pork-based, was significantly higher than that of the kibble diet in both calculations (FDR-adjusted *P* < 0.001 for all). The %ME/gross energy of diet values were also significantly higher in all fresh diets as compared to the kibble diet (FDR-adjusted *P* < 0.001 for all).

**Table 4.** Metabolizable energy (ME, mean ± *SD*) of the study diets

Measure*	Unit	Kibble (n = 12)	Fresh C (n = 12)	Fresh B (n = 6)	Fresh P (n = 6)	Fresh T (n = 6)	P**
Calculated ME	kcal/g FW diet	4.00±0.12^a^	1.41±0.02^b^	1.41±0.01^b^	1.15±0.01^c^	1.45±0.01^d^	2.05E–07
	kcal/g DM diet	4.23±0.13^a^	5.19±0.06^b^	4.98±0.05^c^	4.37±0.03^a^	5.01±0.02^c^	2.31E–07
	%ME/GE	80.8±2.4^a^	87.3±1.0^b^	87.3±0.8^b,d^	85.4±0.5^c^	86.4±0.4^d^	1.27E–06
Measured ME	kcal/g FW diet	3.92±0.14^a^	1.37±0.02^b^	1.41±0.01^c^	1.07±0.01^d^	1.41±0.01^c^	1.47E–07
	kcal/g DM diet	4.14±0.15^a^	5.07±0.07^b^	4.95±0.05^c^	4.07±0.02^a^	4.88±0.03^d^	3.22E–07
	%ME/GE	78.7±2.9^a^	86.4±1.2^b,c^	86.8±0.9^b^	84.0±0.5^d^	85.7±0.5^c^	1.29E–06
Atwater ME	kcal/g FW diet	4.25	1.40	1.40	1.15	1.45	–
	kcal/g DM diet	4.49	5.17	4.94	4.34	4.99	–
	%ME/GE	85.9	87.1	86.7	84.9	86.1	
Modified Atwater ME	kcal/g FW diet	3.83	1.28	1.27	1.03	1.31	–
	kcal/g DM diet	4.05	4.72	4.49	3.89	4.53	–
	%ME/GE	77.4	79.4	78.7	76.1	78.3	
NRC 2006 ME (CF)	kcal/g FW diet	3.90	1.32	1.34	1.09	1.39	–
	kcal/g DM diet	4.12	4.88	4.73	4.14	4.78	–
	%ME/GE	78.8	82.2	82.9	80.9	82.6	
NRC 2006 ME (TDF)	kcal/g FW diet kcal/g DM diet	3.79	1.14	–	–	–	–
		4.01	4.21	–	–	–	–
	%ME/GE	76.6	70.9	–	–	–	

FW: fresh weight, DM: dry matter, GE: gross energy (of food intake), NRC: National Research Council, CF: crude fiber, TDF: total dietary fiber, fresh C: fresh chicken, fresh B: fresh beef, fresh P: fresh pork, fresh T: fresh turkey.

*Calculated ME is calculated from calculated gross energy. Measured ME is calculated from gross energy measured by the bomb calorimetry. Atwater, modified Atwater, and NRC 2006 ME calculations are described in Supplemental Table 1.

**Kruskal–Wallis rank sum test. Means not sharing the same superscript are significantly different (pairwise Wilcoxon rank sum tests with false discovery rate adjustment).

### Differential ME Calculations

Additionally, ME was estimated based on the macronutrient composition (Table 1) using the Atwater calculation, the modified Atwater calculation, or the NRC 2006 calculation for each diet (**Supplemental Table 2**). As shown in Table 4, ME predicted by the NRC 2006 calculation using the CF value (4.12 kcal/g DM) was the closest to the measured ME of the kibble diet (4.14 ± 0.15 kcal/g DM). On the other hand, ME predicted by the Atwater calculation (chicken: 5.17 kcal/g DM, Beef: 4.94 kcal/g DM) was almost identical to the measured ME value for the chicken- and beef-based fresh diets (chicken: 5.07 ± 0.07 kcal/g DM, beef: 4.95 ± 0.05 kcal/g DM). Like the kibble diet, the NRC 2006 calculation with CF value (4.14 kcal/g DM) was closest to the measured ME for the pork-based fresh diet (4.07 ± 0.02 kcal/g DM). The Atwater calculation (4.99 kcal/g DM) and the NRC 2006 calculation with CF (4.78 kcal/g DM) performed comparably in predicting the measured ME of the turkey-based fresh diet (4.88 ± 0.03 kcal/g DM), although the Atwater calculation overestimated and the NRC calculation underestimated the measured ME. The modified Atwater calculation performed worse than either the Atwater calculation or the NRC 2006 calculation with CF for all of the fresh diets, but it performed better than the Atwater calculation for the kibble diet.

## Discussion

This preliminary study is among the first to demonstrate that the apparent total tract nutrient digestibility (DM, protein, fat, NFE, calorie) of commercial fresh dog foods is significantly greater than that of a nutritionally comparable extruded kibble regardless of the macronutrient composition. The fresh diets used in this study had a wide range of macronutrients (fat: 15–30% DM, protein: 37–40% DM, NFE: 24–35% DM) which were similar to the kibble diet (fat: 19% DM, protein: 37% DM, NFE: 31% DM), although the CF and TDF contents in the kibble diet (CF: 3.5% DM, TDF: 13.2% DM) were higher than the fresh diets (CF: 1.0–1.8% DM for all recipes, TDF: 20.3% DM for the chicken-based). Nutrient and calorie digestibilities of extruded kibble, or ingredients commonly used in kibble diets, are much better understood since the major portion of household dogs in the United States are fed kibble diets (Laflamme *et al.*, 2008; Dinallo *et al.*, 2017). The limited knowledge of digestibility of a fresh diet may be counterintuitive since domesticated dogs have been sharing foods with humans for far longer than the invention and widespread adoption of extruded dry kibble.

A canine diet is composed of many factors that may affect nutrient digestibility and ME such as ingredient selection, macronutrient composition, and the amount of dietary fibers (Earle *et al.*, 1998). For example, the digestibility of two extruded kibble diets, one of which composed of chicken by-product meal (commonly used in extruded kibble diets) whereas the other composed of cooked fresh poultry, was compared (Murray *et al.*, 1998). The digestibility of DM, fat, and crude protein in the small intestine (but not total tract) was significantly higher in the fresh poultry diet by 10.0%, 8.9%, and 4.4%, respectively. In a second trial, when a group of dogs switched from a diet containing 0.6% DM to 14.7% DM of CF, the digestibility of DM was reduced from 90% to 70% (Burrows *et al.*, 1982). Likewise, replacing corn with up to 300 g/kg DM potato starch in a kibble diet lowered the amount of CF by 21%, increased the digestibility of DM by 2.4%, calories by 2.0%, and the ME by 0.24 kcal/g (Domingues *et al.*, 2019). When two diets of similar fiber content were fed to a group of adult dogs, the diet that was higher in fat and protein contents led to an increase in digestibility of fat, protein, and calorie (Kienzle *et al.*, 2001). The fresh diets in our study have higher fat (except the pork-based diet) and lower fiber contents than the kibble diet, both of which may synergistically contribute to the significantly higher nutrient and calorie digestibility. Using the precision-fed cecectomized rooster assay, the DM digestibility of six commercial fresh diets has been reported to range from 64.4% to 80.7% (Oba *et al.*, 2020). In a feeding trial, two fresh diets were reported to have DM digestibility of 84.1 ± 1.6% and 85.1 ± 1.5% (Algya *et al.*, 2018), while the average DM digestibility of the four fresh diets used in this study were all greater than 90%.

Given its very short processing duration, extrusion cooking was traditionally thought to preserve nutrients and increase the digestibility of the diet (Singh *et al.*, 2007, Tran *et al.*, 2008). However, emerging evidence demonstrates that it may decrease nutrient bioavailability. For example, extrusion may enhance the formation of a lipid–amylose complex, which inhibits carbohydrate digestion by amylases (Murray *et al.*, 1998). Destruction and racemization of amino acids at very high temperature (Tran *et al.*, 2008), as well as significant loss of amino acids from the Maillard reaction have also been measured after extrusion (Singh *et al.*, 2007). On the other hand, heat cooking (at lower temperature than extrusion cooking) is known to increase nutrient and calorie digestibilities in human diets, especially for plant-based ingredients (Carmody and Wrangham, 2009). We are not aware of any study comparing the digestibility of raw and cooked canine diets with comparable macronutrient composition. In one feeding trial, the digestibility of crude protein and NFE increased after cooking the starch portion of the diet (Kienzle *et al.*, 2001), however this is not always observed across diets with varying CF content. In another feeding trial where one kibble and two fresh diets were compared for their apparent total tract digestibility, fresh diets were shown to have significantly higher digestibilities of crude protein, fat, and calorie, but not DM, than a kibble diet (Algya *et al.*, 2018), although this may reflect the higher protein content of the fresh diets. We similarly observed higher digestibilities of fat, protein, and calories across all fresh diets with a comparable range of macronutrient compositions to the kibble diet in our study (Table 1).

The estimation of ME, defined as the net energy available for growth and maintenance, currently remains largely unknown for fresh diets. An accurate calculation is critical to provide the intended calories for the target animal and because nutrient requirements are sometimes assessed following correction for caloric density. The AAFCO manual allows pet food companies to label the ME by either estimation using modified Atwater factors on the macronutrient composition of the diet or through a testing protocol (direct measurement). The modified Atwater calculation, despite being simple to use, has been shown to underestimate the ME of highly digestible diets and overestimate ME of those with lower digestibility (Laflamme, 2001). The NRC 2006 calculation, on the other hand, takes into account the TDF or CF content, and was demonstrated to estimate the value ME of extruded dry and canned dog diets in 207 feeding trials with higher accuracy than the modified Atwater calculation (Calvez *et al.*, 2019). In the current study we similarly observed that the modified Atwater calculation tended to underestimate the ME and the NRC 2006 estimation was the most accurate predictor of ME for the kibble diet. On the other hand, the standard Atwater calculation performed best for the two fresh diets that had the highest fat content (chicken and beef) while the NRC 2006 calculation performed best for the fresh diets that had the highest protein content (pork). ME of the turkey-based fresh diet was equally overestimated and underestimated with the standard Atwater and NRC 2006 methods, respectively, possibly because of the similar fat content but higher protein content than the beef- and chicken-based diets. Therefore, we propose that commercial and home-prepared fresh diets should be assessed using standard Atwater factors as commonly done in human nutrition, or preferably for commercial products, by direct measurement in conforming feeding trials.

Although similar DM intake was observed during the two chicken-based diets, we observed a significant decrease in fecal DM and defecation frequency during the fresh diet than the kibble diet. This finding confirms observations anecdotally reported from many dog owners, but it contradicts the finding in another study where fecal DM was comparable between the kibble and fresh diets (Algya *et al.*, 2018). Moreover, there was also a trend towards lower weight gain during the chicken-based fresh diet period than the chicken-based kibble diet period, while GE intake during the two feeding periods was comparable and the ME of the fresh diet was significantly higher than the kibble. Increased energy expenditure through physical activity may explain this observation. Food with higher moisture was shown to increase total physical activity in cats (Deng *et al.*, 2014), but not in dogs (though physical activity was recorded for only 24 h) (Algya *et al.*, 2018). The chicken-based fresh diet used in this study has much higher moisture than the kibble diet (72.90% fresh weight vs. 5.40% fresh weight). Likewise, food with higher moisture content led to lower weight gain in cats even with comparable caloric intake across the diets (Cameron *et al.*, 2011).

Although the novelty of this study lies mainly in the determination of nutrient digestibility and ME in commercial fresh diets with a digestibility trial, some limitations need to be addressed. A difference in the calorie intake among the test diets was likely to explain the significant body weight change in the kibble and pork-based diets and may confound any observed results (Table 2 and **Supplemental Table 3**), although this difference in the intake has been mathematically adjusted in the digestibility calculation. An attempt was made to match the test diets for similar nutrient compositions and ingredients, but due to the nature of these diets there was some variability of nutritional profiles (Table 1). The impact of each individual nutritional component cannot also be assessed in this current study, but our findings reflect a combination of nutrients in each diet that may synergistically affect the digestibility and the GM composition. Results reported in this study are also specific to the test diets and cannot be extrapolated to other brands or formulations, and larger studies with improved design, such as randomized controlled or cross-over trials, will aid in validation of these findings. However, as a growing number of consumers choose to feed fresh diets to their companion dogs, the results can have a direct impact on household dogs.

## Supplementary Material

txab071_suppl_Supplementary_Table_S1

txab071_suppl_Supplementary_Table_S2

txab071_suppl_Supplementary_Table_S3

txab071_suppl_Supplementary_Table_S4
