# Supplementary material for: Apparent total tract nutrient digestibility and metabolizable energy estimation in commercial fresh and extruded dry kibble dog foods
Source: Transl Anim Sci. 2021 May 27;5(3):txab071. doi: 10.1093/tas/txab071 (PMC8279163; doi:10.1093/tas/txab071)
Supplement: txab071_suppl_Supplementary_Table_S1 [file txab071_suppl_supplementary_table_s1.docx]

**Supplementary Table 1.** List of fresh diet ingredients

| **Fresh diet** | **Ingredients** |
| --- | --- |
| Chicken Chow | Diced chicken, sweet potatoes, yellow squash, spinach, sunflower oil, dicalcium phosphate, canola oil, calcium carbonate, fish oil, vinegar, citric acid, taurine, choline bitartrate, zinc gluconate, ferrous sulfate, vitamin E supplement, copper gluconate, manganese gluconate, thiamine mononitrate (vitamin B1), selenium yeast, riboflavin (vitamin B2), vitamin B12 supplement, cholecalciferol (vitamin D3), potassium iodide. |
| Beef Mash | Ground beef, russet potatoes, eggs, carrots, peas, dicalcium phosphate, calcium carbonate, salt, fish oil, sunflower oil, vinegar, citric acid (preservative), taurine, choline bitartrate, zinc gluconate, ferrous sulfate, vitamin E supplement, copper gluconate, manganese gluconate, thiamine mononitrate (vitamin B1), selenium yeast, riboflavin (vitamin B2), vitamin B12 supplement, cholecalciferol (vitamin D3), potassium iodide. |
| Pork Potluck | Ground pork, russet potatoes, green beans, yellow squash, brown mushrooms, kale, dicalcium phosphate, salt, fish oil, vinegar, citric acid, taurine, choline bitartrate, zinc gluconate, ferrous sulfate, vitamin E supplement, copper gluconate, manganese gluconate, thiamine mononitrate (vitamin B1), selenium yeast, riboflavin (vitamin B2), vitamin B12 supplement, cholecalciferol (vitamin D3), potassium iodide. |
| Turkey Fare | Ground turkey, eggs, brown rice, carrots, spinach, dicalcium phosphate, calcium carbonate, salt, fish oil, vinegar, citric acid, taurine, choline bitartrate, zinc gluconate, ferrous sulfate, vitamin E supplement, copper gluconate, manganese gluconate, thiamine mononitrate (vitamin B1), selenium yeast, riboflavin (vitamin B2), vitamin B12 supplement, cholecalciferol (vitamin D3), potassium iodide. |
