# Supplementary material for: Apparent total tract nutrient digestibility and metabolizable energy estimation in commercial fresh and extruded dry kibble dog foods
Source: Transl Anim Sci. 2021 May 27;5(3):txab071. doi: 10.1093/tas/txab071 (PMC8279163; doi:10.1093/tas/txab071)
Supplement: txab071_suppl_Supplementary_Table_S2 [file txab071_suppl_supplementary_table_s2.docx]

**Supplemental Table 2.** Food digestibility and energy calculation

| **Measure** | **Calculation** |
| --- | --- |
| Dry Matter (DM) Digestibility | [{(Daily Food Consumed in g) x (% DM of Food)} — {(Daily Weight of Stool in g) x (% DM of Stool)}] ÷ {(Daily Food Consumed in g) x (% DM of Food)} |
| Protein Digestibility | [{(Daily Food Consumed in g) x (% Protein of Food)} — {(Daily Weight of Stool in g) x (% Protein of Stool)}] ÷ {(Daily Food Consumed in g) x (% Protein of Food)} |
| Fat Digestibility | [{(Daily Food Consumed in g) x (% Fat of Food)} — {(Daily Weight of Stool in g) x (% Fat of Stool)}] ÷ {(Daily Food Consumed in g) x (% Fat of Food)} |
| Caloric Digestibility | [{(Daily Food Consumed in g) x (Gross Energy of Diet in kcal/g)} — {(Daily Weight of Stool in g) x (Gross Energy of Stool in kcal/g)}] ÷ {(Daily Food Consumed in g) x (Gross Energy of Diet in kcal/g)} |
| Calculated Gross Energy (GE) of Diet | (9.4 kcal/g x Crude Fat in g) + (5.65 kcal/g x CP in g) + (4.15 kcal/g x NFE in g) |
| Calculated Gross Energy (GE) of Stool | (9.4 kcal/g x Fecal Fat in g) + (5.65 kcal/g x Fecal Protein in g) + (4.15 kcal/g x Fecal NFE in g) |
| Nitrogen-Free Extract (NFE) | 100 — (% CP+ % Crude Fat + % CF + % Moisture + % Ash) |
| Metabolizable Energy (ME) | {Gross Energy of Diet in kcal — Gross Energy of Stool in kcal — (Grams Protein Digested in g x 1.25 kcal/g)} ÷ Amount of Food Consumed in g or kcal |
| Atwater ME | [(4 kcal/g x CP in g) + (9 kcal/g x Crude Fat in g) + (4 kcal/g x NFE in g)] ÷ Amount of Food Consumed in g |
| Modified Atwater ME | [(3.5 kcal/g x CP in g) + (8.5 kcal/g x Crude Fat in g) + (3.5 kcal/g x NFE in g)] ÷ Amount of Food Consumed in g |
| National Research Council 2006 ME using CF | GE in kcal = (9.4 kcal/g x Crude Fat in g) + (5.65 kcal/g x CP in g) + (4.15 kcal/g x NFE in g)  Energy Digestibility in % = 91.2.6 - (1.43 x CF in %DM)  Digestible Energy in kcal = (GE in kcal x Energy Digestibility in %)/100  ME in kcal/g = {Digestible Energy in kcal - (1.04 kcal/g x CP in g )}/Food Intake in g |
| National Research Council 2006 ME using TDF | GE in kcal = (9.4 kcal/g x Crude Fat in g) + (5.65 kcal/g x CP in g) + (4.15 kcal/g x NFE in g)  Energy Digestibility in % = 96.6 - (0.95 x TDF in %DM)  Digestible Energy in kcal = (GE in kcal x Energy Digestibility in %)/100  ME in kcal/g = {Digestible Energy in kcal - (1.04 kcal/g x CP in g )}/Food Intake in g |

CP: crude protein, CF: crude fiber, TDF: total dietary fiber
