# Supplementary material for: Apparent total tract nutrient digestibility and metabolizable energy estimation in commercial fresh and extruded dry kibble dog foods
Source: Transl Anim Sci. 2021 May 27;5(3):txab071. doi: 10.1093/tas/txab071 (PMC8279163; doi:10.1093/tas/txab071)
Supplement: txab071_suppl_Supplementary_Table_S3 [file txab071_suppl_supplementary_table_s3.docx]

**Supplemental Table 3.**  Total weight change (mean±SD) during each 10-day feeding period (n=12)

| **Measure** | **Kibble**  **(n=12)** | **Fresh C**  **(n=12)** | **Fresh B**  **(n=6)** | **Fresh P (n=6)** | **Fresh T (n=6)** | **p value*** |
| --- | --- | --- | --- | --- | --- | --- |
| Weight at day 1 of each diet period, in kg  Total body weight change, in kg  Total body weight change, in % | 10.88±1.40  0.40±0.22^a,†^  3.8±2.1^a^ | 11.30±1.43  0.15±0.24^b^  1.5±2.2^a,b^ | 10.52±2.29  -0.07±0.20^b,c^  -0.4±2.4^b,c^ | 12.35±1.52  -0.32±0.13^c,†^  -2.5±0.8^c^ | 11.60±1.56  -0.15±0.16^c^  -1.2±1.3^c^ | 0.412  3.10E-05  5.75E-05 |

FW: fresh weight, DM: dry matter, Fresh C: Fresh Chicken, Fresh B: Fresh Beef, Fresh P: Fresh Pork, Fresh T: Fresh Turkey

*Kruskal-Wallis rank sum test. Means not sharing the same superscript are significantly different (pairwise Wilcoxon rank sum tests with false discovery rate adjustment, adjusted p<0.05).

^†^ Wilcoxon signed-rank test shows body weight on day 10 was significantly different from day 1 within each diet (p<0.05).
