# Supplementary material for: Apparent total tract nutrient digestibility and metabolizable energy estimation in commercial fresh and extruded dry kibble dog foods
Source: Transl Anim Sci. 2021 May 27;5(3):txab071. doi: 10.1093/tas/txab071 (PMC8279163; doi:10.1093/tas/txab071)
Supplement: txab071_suppl_Supplementary_Table_S4 [file txab071_suppl_supplementary_table_s4.docx]

**Supplemental Table 4.** Fecal frequency and consistency (mean±SD) during each feeding period

| **Measure** | **Kibble**  **(n=12)** | **Fresh C**  **(n=12)** | **Fresh B**  **(n=6)** | **Fresh P**  **(n=6)** | **Fresh T**  **(n=6)** | **p value*** |
| --- | --- | --- | --- | --- | --- | --- |
| Daily fecal frequency  Fecal consistency score** | 1.7±0.5^a^  3.4±0.2^a,b^ | 1.2±0.2^b^  3.5±0.1^a^ | 1.1±0.2^b,c^  3.4±0.2^a,b^ | 1.1±0.1^b,c^  3.4±0.1^a,b^ | 1.0±0.2^c^  3.1±0.2^b^ | 6.50E-04  0.020 |

Fresh C: Fresh Chicken, Fresh B: Fresh Beef, Fresh P: Fresh Pork, Fresh T: Fresh Turkey

*Kruskal-Wallis rank sum test. Means not sharing the same superscript are significantly different (pairwise Wilcoxon rank sum tests with false discovery rate adjustment).

**Average fecal consistency score was calculated from all samples from day 6 (PM) to day 11 (AM) of each diet period. Fecal score ranged from 1: watery diarrhea; 1.5: diarrhea; 2: moist, no form; 2.5: moist, some form; 3: moist, formed; 3.5: well formed, sticky; 4: well formed; 4.5: hard, dry; 5: hard, dry, crumbly.
